# TRAJECTORIES OF C-REACTIVE PROTEIN OVER THE MENOPAUSE TRANSITION: THE STUDY OF WOMEN’S HEALTH ACROSS THE NATION

**DOI:** 10.1093/geroni/igad104.1482

**Published:** 2023-12-21

**Authors:** Samar El Khoudary, Meiyuzhen Qi, Xirun Chen, Gail Greendale, Arun Karlamangla, Karen Matthews

**Affiliations:** University of Pittsburgh School of Public Health, Pittsburgh, Pennsylvania, United States; University of Pittsburgh School of Public Health, Pittsburgh, Pennsylvania, United States; University of Pittsburgh School of Public Health, Pittsburgh, Pennsylvania, United States; UCLA School of Medicine, Los Angeles, California, United States; University of California, Los Angeles, Los Angeles, California, United States; University of Pittsburgh School of Public Health, Pittsburgh, Pennsylvania, United States

## Abstract

Inflammation is a mechanistic pillar of aging contributing to the pathogenesis of several common medical conditions. Numerous in-vitro and animal experiments support a link between estrogen decline over the menopause transition (MT) and inflammation. Whether the MT per-se is associated with increased inflammatory load is not obvious. Using longitudinal data from 1,459 (11,373 observations) women (at baseline: 42-52 years old; underweight/normal 39.6%, overweight 28%, obese 32.4%; White 44.5%, Black 27.8%, Chinese 10.7%, Japanese 12.1%, Hispanic 4.9%) with a known final-menstrual-period (FMP) date and at least 3-measures of high sensitivity C-reactive protein (hs-CRP) spanning between 10 years before to 14 years after FMP, we characterized trajectories of hs-CRP and their associations with premenopausal obesity and race/ethnicity. Observations indicating acute inflammation were deleted and data were censored at first use of hormone therapy. Group-based trajectory modeling adjusted for time-varying smoking-status and steroids/statin use was utilized to identify trajectories. LOESS plots and piecewise linear mixed-effects models were used to determine if trajectories were linear or had inflection points around FMP. Three distinct hs-CRP trajectories were identified: low-rise (low-level significantly increased 2.5Yrs after FMP; 26.6%), medium-stable (remained stable across MT; 41.7%), and high-decline (high-level significantly declined at FMP; 31.7%). White women were more likely to show the medium-stable trajectory, Black and Hispanic the high-decline, and Chinese and Japanese the low-rise trajectory. Premenopausal obesity predicted the high-decline trajectory, underweight/normal predicted the low-rise, while overweight predicted the medium-stable trajectory. Heterogeneous patterns of hs-CRP over the MT exist and are related to women’s race/ethnicity and premenopausal obesity.

